# Task-dependent recruitment of intrinsic brain networks reflects normative variance in cognition

**DOI:** 10.1002/brb3.243

**Published:** 2014-07-09

**Authors:** Jennifer L Gess, Jennifer S Fausett, Tonisha E Kearney-Ramos, Clinton D Kilts, George Andrew James

**Affiliations:** Psychiatric Research Institute, University of Arkansas for Medical SciencesLittle Rock, Arkansas, 72205-7199

**Keywords:** Cognition, Cognitive Connectome, fMRI, functional neuroimaging, individual differences, neuropsychology

## Abstract

**Background:**

Functional neuroimaging has great potential to inform clinical decisions, whether by identifying neural biomarkers of illness progression and severity, predicting therapeutic response, or selecting suitable patients for surgical interventions. Yet a persisting barrier to functional neuroimaging's clinical translation is our incomplete understanding of how normative variance in cognition, personality, and behavior shape the brain's structural and functional organization. We propose that modeling individual differences in these brain–behavior relationships is crucial for improving the accuracy of neuroimaging biomarkers for neurologic and psychiatric disorders.

**Methods:**

We addressed this goal by initiating the Cognitive Connectome Project, which bridges neuropsychology and neuroimaging by pairing nine cognitive domains typically assessed by clinically validated neuropsychological measures with those tapped by canonical neuroimaging tasks (motor, visuospatial perception, attention, language, memory, affective processing, decision making, working memory, and executive function). To date, we have recruited a diverse sample of 53 participants (mean [SD], age = 32 [9.7] years, 31 females).

**Results:**

As a proof of concept, we first demonstrate that our neuroimaging task battery can replicate previous findings that task performance recruits intrinsic brain networks identified during wakeful rest. We then expand upon these previous findings by showing that the extent to which these networks are recruited by task reflects individual differences in cognitive ability. Specifically, performance on the Judgment of Line Orientation task (a clinically validated measure of visuospatial perception) administered outside of the MRI scanner predicts the magnitude of task-induced activity of the dorsal visual network when performing a direct replication of this task within the MRI scanner. Other networks (such as default mode and right frontoparietal) showed task-induced changes in activity that were unrelated to task performance, suggesting these networks to not be involved in visuospatial perception.

**Conclusion:**

These findings establish a methodological framework by which clinical neuropsychology and functional neuroimaging may mutually inform one another, thus enhancing the translation of functional neuroimaging into clinical decision making.

## Introduction

The use of functional magnetic resonance imaging (fMRI) to map the neurobiological correlates of behavior, emotion, and cognition has rapidly grown over the past two decades (Ogawa et al. 1993; Cabeza and Nyberg 2000; Smith et al. 2009). Yet fMRI research has predominantly focused upon group-level neural representations of cognition, with surprisingly little emphasis placed upon how the brain encodes normative variance in cognition; as evidence, less than 2% of journal articles indexed by PubMed with the search term “functional MRI” or “fMRI” also included the search term “individual differences.” Our incomplete understanding of how brain function is shaped by normative variance in cognition, personality, and behavior remains a persisting barrier to the clinical translation of fMRI. For example, an exaggerated amygdala response to negatively valent images (e.g., sad or fearful faces) is a hallmark characteristic of depression (Fu et al. 2004; Leppanen 2006; Lee et al. 2007; Stuhrmann et al. 2013) but has also been associated in nonclinical populations with neuroticism (Stein et al. 2007) and trait anxiety (Hare et al. 2008; Ewbank et al. 2009). The high comorbidity of neuroticism and anxiety with depression (Roy 1990; Maier et al. 1992; Beautrais et al. 1999; Bienvenu et al. 2001; Jylha et al. 2009) obviates the need to understand how personality traits influence brain function in normative and clinical populations, lest our search for neural biomarkers of depression uncover biomarkers of neuroticism. The neuroimaging research literature is replete with similar examples for other clinical disorders, arguing that the mapping of brain function to normative cognitive variance is necessary before functional neuroimaging findings can be meaningfully translated into patient-oriented clinical decision making.

To address this need, we have initiated the Cognitive Connectome Project, a merging of clinically validated neuropsychological tests, personality assessments, and canonical fMRI tasks purported to assess similar cognitive domains. The Cognitive Connectome evaluates cognition across nine domains: motor, visuospatial, attention, language and cognitive fluency, memory, affective processing, decision making and reward processing, working memory, and executive function. These assessments of cognitive function include direct replications of neuropsychological evaluations within the MRI scanner when available and/or feasible (such as the Judgment of Line Orientation Task) as well as conceptual replications (such as the D-KEFS Tower task outside the scanner and Tower of London task inside the scanner). By focusing on neuroimaging tasks inspired by age-normed neuropsychological instruments that are broadly used for clinical evaluation of cognitively impaired populations, we seek to (1) map the neural representation of individual differences in cognitive ability across defined behavioral domains (Fox et al. 2005), and (2) initiate the development of an interpretive framework for the translation of functional neuroimaging into clinical care settings.

As a demonstration of the Cognitive Connectome's utility, we sought to replicate previous findings that intrinsic brain networks identified during wakeful rest are also recruited during task demands. We then expanded upon these findings by modeling how individual differences in cognitive ability influence the degree to which tasks recruit brain networks, an advantage the Cognitive Connectome offers over group-derived meta-analytic approaches. Overall, we sought to characterize the neural correlates of individual differences in cognitive ability by studying a sample of carefully screened and characterized healthy adults to initiate a functional connectome of eventual application to the neuroscience of individual clinical patients with disorders of cognition.

## Methods

### Participants

#### Demographics

Participants were recruited from community advertisements in accordance with University of Arkansas for Medical Sciences (UAMS) Institutional Review Board approval and oversight. Fifty-three participants (mean [SD], age = 32 [9.7] years; range = 19–50 years; 31 female, 22 male; 22 self-reporting as African-American, 31 Caucasian, 1 Hispanic) consented to participate in the study, met inclusion and exclusion criteria, and completed at least one study session. Sample demographics are provided in Table 1. Inclusion criteria for this study were healthy men and women, aged 18–50 years, without histories of psychiatric or neurologic illness, and who were native English speakers with at least an eighth grade reading and writing proficiency. Exclusion criteria were presence of DSM-IV psychiatric disorders as determined by structured clinical interview (SCID-I NP), self-reported history of neurological disorders or loss of consciousness exceeding 10 min, substance abuse or dependence (excluding nicotine dependence), and contraindications to the high-field MRI environment such as ferromagnetic implants (determined through self-reported medical history and screening with the SAFESCAN® Target Scanner™, Mednovus, Inc., Escondido, CA) and pregnancy (determined through urinalysis).

**Table 1:** Participant demographics

Number of participants	53
Age (years)	
Mean (SD)	32 (9.7)
Range	19–50
Sex, *n* (%)	
Female	31 (58)
Male	22 (42)
Ethnicity, *n* (%)	
African American	22 (42)^1^
Caucasian	31 (58)^1^
Hispanic/Latino	1 (2)
Terminal education, *n* (%)	
Grade 7–12 (without graduation)	3 (6)
High school or certificate of high school equivalency	5 (9)
Partial college or currently enrolled	20 (38)
Graduation from 2-year college	4 (8)
Graduation from 4-year college	7 (13)
Partial graduate/Professional school	8 (15)
Degree from graduate/Professional school	6 (11)
Handedness, *n* (%)	
Left	6 (11)
Right	45 (85)
Unreported	2 (4)

1 Includes one participant self-reporting as both African American and Caucasian.

#### Recruitment and procedures

All procedures were performed at the Brain Imaging Research Center (BIRC) of the Psychiatric Research Institute at UAMS. Participants first underwent a brief telephone interview to determine eligibility. Eligible participants were invited to the BIRC, where they provided written informed consent to participate in the study, followed by the SCID-NP and medical history (1 h) to determine if participants met exclusion criteria. Eligible participants then underwent a battery of computerized assessments (1 h), two MRI sessions (1 h each; session order was randomly counterbalanced across participants), and comprehensive neuropsychological assessment (2–4 h), scheduled at the participants' convenience across 1–4 sessions. Urinalysis was conducted prior to each MRI session to determine pregnancy or illicit drug use (both exclusionary criteria). Table 2 provides a full list of neuroimaging tasks and testing batteries for each cognitive domain, with full descriptions in the *Supporting Information*. A measure of general intelligence (such as IQ) was not administered due to time constraints, since this measure can be derived via factor analysis of the administered tests.

**Table 2:** Cognitive connectome tasks and instruments

Cognition/Modality	Neuropsychological assessments	fMRI tasks
Motor	Grooved Pegboard Halstead–Reitan Finger-Tapping Test	Finger-Tapping Task
Visuospatial	Judgment of Line Orientation Task Rey–Osterrieth Complex Figure (copy)	Judgment of Line Orientation Task Flashing Checkerboard Task
Attention	Test of Everyday Attention (TEA) Digit Span (WAIS-IV): forward subtest Spatial Span (WMS-III): forward subtest	n-back (0-back condition)
Language and cognitive fluency	D-KEFS Verbal Fluency Boston Naming Task	Letters and Category Verbal Fluency (Controlled Oral Word Association Task)
Memory	Verbal Paired Associates Task (WMS-IV) California Verbal Learning Test Brief Visuospatial Memory Test Revised	Verbal Paired Associates Task Encoding International Affective Picture System (IAPS) stimuli Recognition of IAPS stimuli
Affective	Emotion Regulation Questionnaire (ERQ)	Rating emotional IAPS pictures
Decision making	Intertemporal Choice Behavior (delayed discounting)	Iowa Gambling Task
Working memory	Digit Span (WAIS-IV): backward, sequence Spatial Span (WMS-III): reverse	n-back (2-back condition)
Metacognition and executive function	D-KEFS Tower Test D-KEFS Color–Word Test Wisconsin Card Sorting Task Booklet Category Test D-KEFS Trails Test D-KEFS Proverbs Test	Tower of London Task Multi-Source Interference Task (MSIT)
Additional individual variables and MRI scans	Big Five Inventory (BFI) Beck Depression Inventory (BDI) Childhood Trauma Questionnaire (CTQ) State-Trait Anxiety Inventory (STAI) Leisure Time Exercise Questionnaire	Resting-state scan (×2) Magnetization prepared rapid acquisition gradient echo (MPRAGE) anatomic scan (×2) Diffusion tensor Imaging

#### Study completion and missing data

The Cognitive Connectome required 6–8 h of assessments per individual. Of the 53 participants who met inclusion and exclusion criteria, all (100%) completed the computerized testing batteries and questionnaires, 46 (87%) completed session A fMRI tasks, 41 (77%) completed session B fMRI tasks, 38 (75%) completed both fMRI sessions, 43 (81%) completed all neuropsychological assessments, and 42 (79%) completed all study assessments. However, performance and feedback from the first five pilot participants (001–007) led to changes in fMRI task designs of the following tasks: Tower of London, n-back, visual affective processing, visual memory, verbal memory, visual checkerboard, and motor tasks. Consequently, these participants' data were not included in the respective task analyses. The analyses below used all available data.

### Neuroimaging

#### Acquisition

Imaging data were acquired using a Philips 3T Achieva X-series MRI scanner (Philips Healthcare, Eindhoven, The Netherlands). Anatomic images were acquired with a MPRAGE sequence (matrix = 256 × 256, 220 sagittal slices, TR/TE/FA = shortest/shortest/8°, final resolution = 0.94 ×0.94 × 1 mm^3^ resolution). Functional images for early participants (001–050) were acquired using an 8-channel head coil with an echo planar imaging (EPI) sequence (TR/TE/FA = 2000 msec/30 msec/90°, FOV=240 × 240 mm, matrix = 80 × 80, 37 oblique slices [parallel to orbitofrontal cortex to reduce sinus artifact], interleaved ascending slice acquisition, slice thickness = 4 mm, final resolution 3.0 × 3.0 × 4.0 mm^3^). For these subjects, session B's resting-state scan was acquired with these parameters but with 3-mm slice thickness, to be consistent with data acquired for other BIRC studies. Functional images for later participants (051+) were acquired using a 32-channel head coil with the following EPI sequence parameters: TR/TE/FA = 2000 msec/30 msec/90°, FOV = 240 × 240 mm, matrix = 80 × 80, 37 oblique slices, ascending sequential slice acquisition, slice thickness = 2.5 mm with 0.5 mm gap, final resolution 3.0 × 3.0 × 3.0 mm^3^. Parameters for the 32-channel coil were selected to reduce orbitofrontal signal loss due to sinus artifact. All analyses included head coil as a covariate of no interest. Diffusion tensor imaging data were also collected (32 directions, TR/TE/FA = 6228 msec/71 msec/90°, FOV = 224 × 224 × 120, matrix = 128 × 128 × 120, final resolution 1.75 × 1.75 × 2.0 mm^3^, 60 slices of 2 mm thickness), and will be discussed in future work.

#### Data preprocessing

All MRI data preprocessing was performed using AFNI (Cox 1996). Anatomic data underwent skull stripping, spatial normalization to the icbm452 brain atlas, and segmentation into white matter (WM), gray matter (GM), and cerebrospinal fluid (CSF) with FSL (Jenkinson et al. 2012). Functional data underwent despiking; slice correction; deobliquing (to 3 × 3 × 3 mm^3^ voxels); motion correction; transformation to the spatially normalized anatomic image; regression of motion parameters, mean timecourse of WM voxels, and mean timecourse of CSF voxels; spatial smoothing with a 6-mm FWHM Gaussian kernel; scaling to percent signal change. Preprocessing scripts are available upon request.

#### Assessing motion artifact

After data preprocessing, independent component analysis (ICA) was used to identify and remove motion-related noise components with the Group ICA of fMRI Toolbox (GIFT v1.3) for Matlab (Calhoun et al. 2001). Head motion artifact manifests in the functional data as alternating “bands” or “stripes” of correlated activity corresponding to the order of slice acquisition. For each functional dataset, ICA solved for the optimal number of components as determined by GIFT's MDL algorithm (typically 100–200 components). Because the pattern of slice acquisition (e.g., all even slices or all odd slices) does not represent biologically plausible brain activity, a liberal threshold (*r* > 0.05) was used to identify components that correlated with slice acquisition. These components were removed from the preprocessed functional data using the “icatb_removeArtifact.m” command in Matlab. Motion artifact was assessed before and after ICA “stripe” removal using single voxel seed-based correlation analyses via AFNI's “InstaCorr” function. Any dataset that continued to have alternating “stripes” of correlated activity were removed from further analysis. All included datasets had less than 3-mm longitudinal movement and less than 3° rotational movement.

#### Independent component analyses

**Figure 1:**
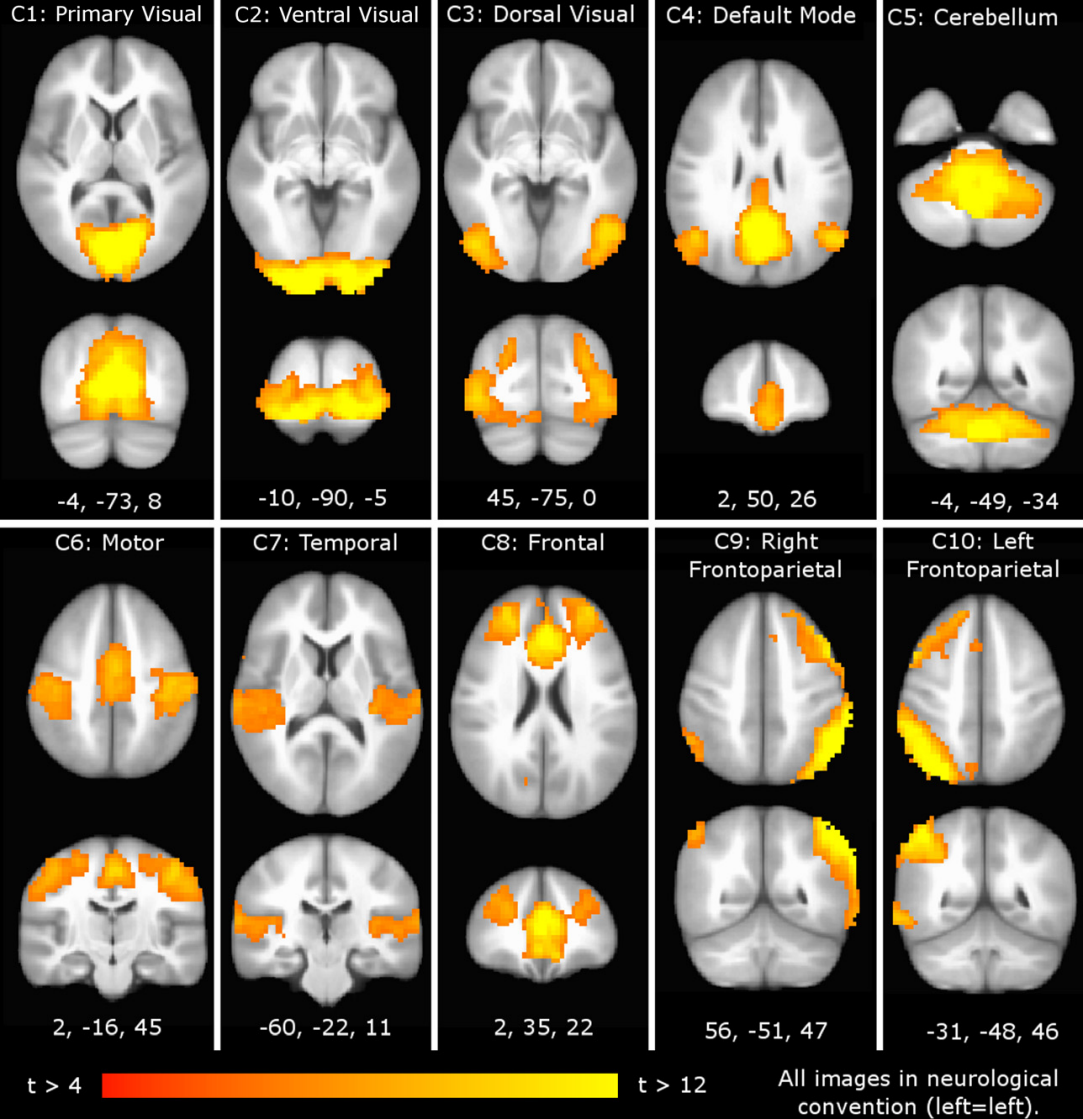
Canonical intrinsic networks identified via ICA. These analyses used the 10 canonical resting-state networks reported by Smith et al. (2009). Component maps depict voxels with positive contributions (*t*-scores ≥4) to each component timecourse. Components are depicted in neurological convention using representative axial and coronal slices at the MNI coordinates provided beneath the coronal image. Top to bottom, left to right: C_1_, primary visual network; C_2_, ventral visual network; C_3_, dorsal visual network; C_4_, default mode network; C_5_, cerebellar network; C_6_, motor network; C_7_: temporal network; C_8_: frontocingulate network; C_9_: right frontoparietal network; C_10_: left frontoparietal network.

Because a goal of this study was to replicate the finding of task-based recruitment of intrinsic brain networks reported previously by Smith et al. (2009), the same networks reported for that analysis were used for this study. As described previously, these networks were derived from ICA of resting-state fMRI data from an independent sample of 36 healthy adults using the FSL program MELODIC; for full details, please see Smith et al. (2009). Smith et al. identified 20 components, with 10 “judged to be artifactual or of more complex interpretation” and discussed only in Supporting Information (Smith et al. 2009). The remaining 10 networks have been well replicated in the literature (Beckmann et al. 2005; Damoiseaux et al. 2006) and were demonstrated to be task recruited; these 10 canonical networks are depicted in Figure 1 and were used for the current analyses.

#### Extracting component activity timecourses

Timecourses of component activity during the functional tasks were generated as follows. First, ICA components were resampled to the postprocessed fMRI data resolution (3 × 3 × 3 mm^3^). Given ICA component X and fMRI dataset Y, the voxelwise dot product was calculated between X and each image (1 − *N*) of Y. Thus, the voxelwise intensities of each image in Y are weighted by that voxel's contribution to component X. This process was repeated for all participants, components, and task fMRI datasets. Figure S1 illustrates this process, which is validated and fully described elsewhere (James et al. 2014).

#### Statistical analyses of tasks

The resulting fMRI timecourses underwent block- or event-related general linear modeling (GLM) as appropriate with AFNI's 3dDeconvolve program (see Supporting Information for deconvolution details; code available upon request). All GLMs incorporated six head motion parameters (x, y, z, roll, pitch, yaw) into the baseline model. GLM yielded a beta value (*β*_M,__N,__P_) and *t*-score (*t*_M,__N,__P_) for each component *X*_M_, task condition *T*_N_, and participant *Z*_P_ that describes the component's task-dependent recruitment for that participant. These component, task condition, and participant-specific *β*s were used for subsequent analyses.

#### Performance-independent task-dependent recruitment

**Figure 2:**
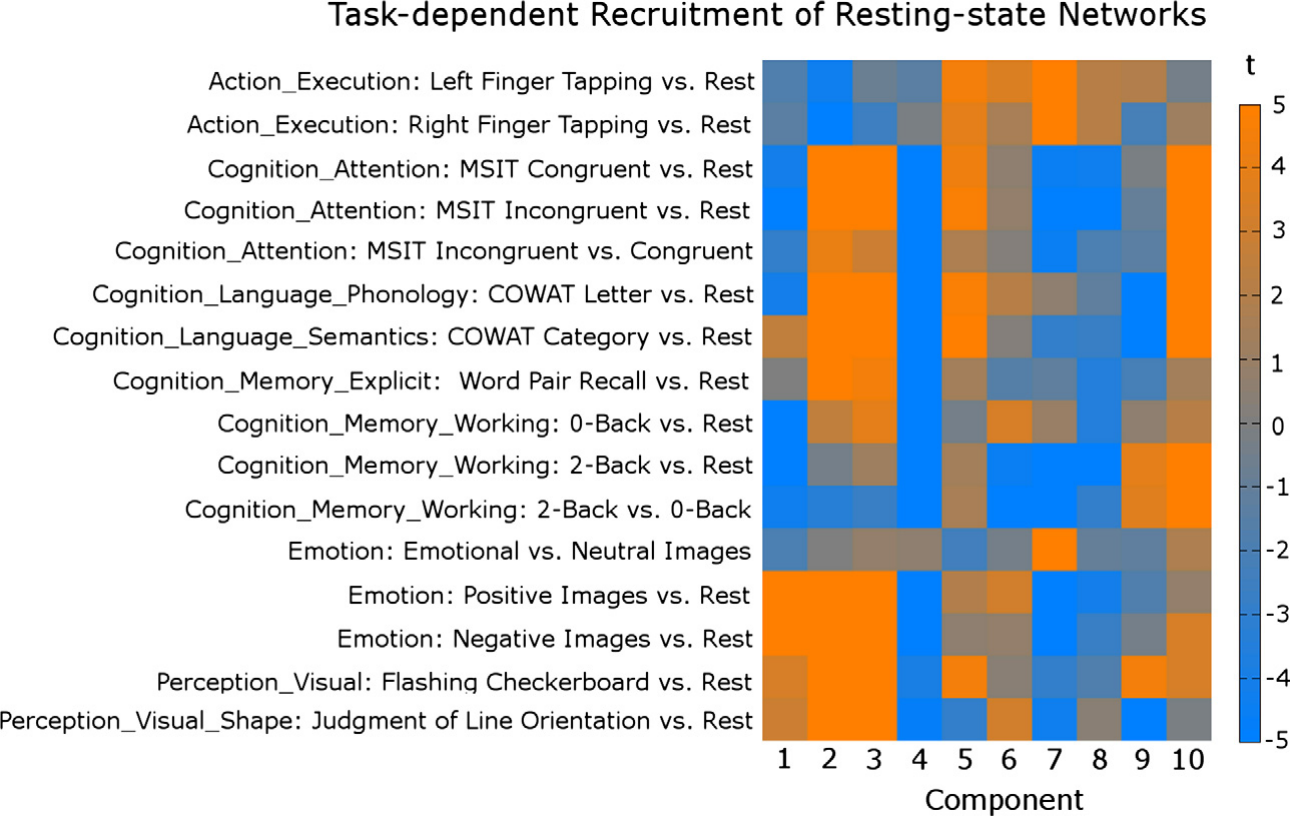
Task-dependent recruitment of intrinsic networks. Task-dependent recruitment of the 10 canonical intrinsic networks was assessed for several Cognitive Connectome fMRI tasks. The ordinate axis provides task contrasts and the BrainMap behavioral domain best matched by each contrast, and the abscissa indicates component/network number. Color coding indicates the significance of contrast-dependent activations (comparing group *β*s against 0), with orange indicating *t* ≥ 5 and blue indicating *t* ≤ −5.

**Figure 3:**
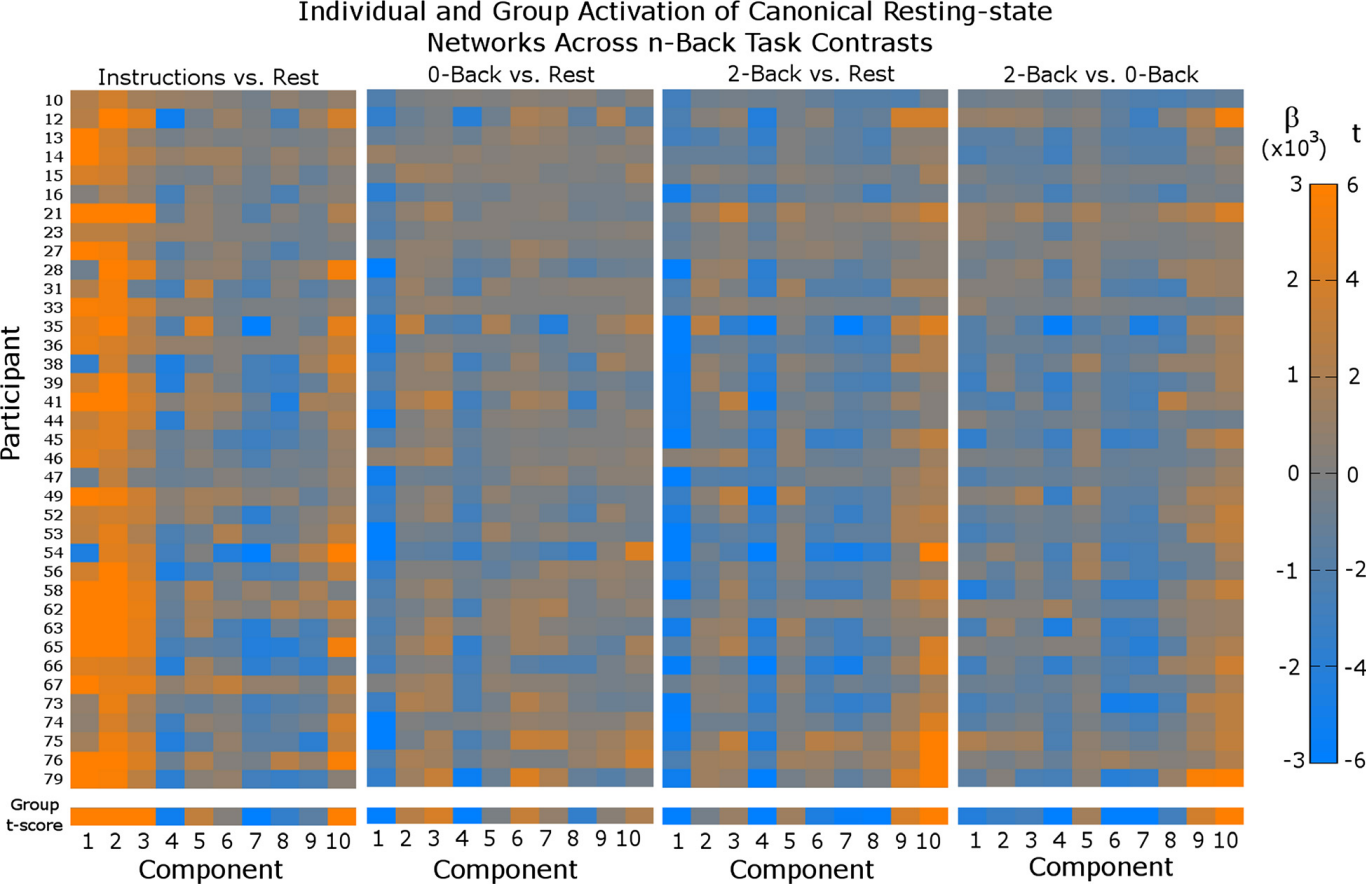
Individual and group activation of canonical intrinsic networks across n-back task contrasts. Component *β* values are depicted for each participant (ordinate axis) and component/network (abscissas) for four n-Back task contrasts, from left to right: Instructions versus Rest, 0-Back versus Rest, 2-Back versus Rest, and 2-Back versus 0-Back. Color coding indicates *β* value magnitude, with orange indicating relative activation for the contrast (*β* ≥ 3 × 10^3^) and blue indicating relative deactivation (*β* ≤ −3 × 10^3^). Group-level significance of activation is also depicted for each network and contrast, with orange indicating *t* ≥ 5 and blue indicating *t* < −5.

For a given component *X*_i_ and task condition *T*_j_, group *t*-tests assessed if participant *β*_i_,_j_ values significantly differed from zero. Figure 2 depicts group-level activation of the canonical resting-state networks across a range of Cognitive Connectome task contrasts, selected as most representative of the following BrainMap behavioral domains: Finger Tapping versus Rest (Action_Execution), MSIT Incongruent versus Congruent (Cognition_Attention), COWAT Letter versus Rest (Cognition_Language_Phonology), COWAT Category versus Rest (Cognition_Language_Semantics), Word Pair Recall versus Rest (Cognition_Memory_Explicit), n-Back 2-Back versus 0-Back (Cognition_Memory_Working), IAPS Emotional versus Neutral Images (Emotion), Flashing Checkerboard versus Rest (Perception_Vision), and Judgment of Line Orientation versus Rest (Perception_Vision_Shape). Similarly, Figure 3 depicts individual variance in participant *β* scores and group *t*-statistics for four n-Back task contrasts and all 10 components.

#### Performance-dependent task-dependent recruitment

The influence of participant performance upon task-dependent recruitment of intrinsic brain networks was assessed as follows. First, stepwise linear regression was conducted to relate the dependent variable (participants' *β* scores for a component and task) to the independent variables of performance (neuropsychological test score), age, sex, education, handedness, head coil, and session order. Statistical thresholds for inclusion and exclusion of independent variables in the stepwise linear regression were *P* < 0.05 and *P* > 0.10, respectively. The Judgment of Line Orientation task (JLO) was analyzed because, of all Cognitive Connectome tasks, its administration as an fMRI task most closely mirrored its administration as a neuropsychological task. Participants' mean duration for JLO trials was also included as a covariate, since brain activity has been shown to increase linearly with longer reaction times (Yarkoni et al. 2009).

**Figure 4:**
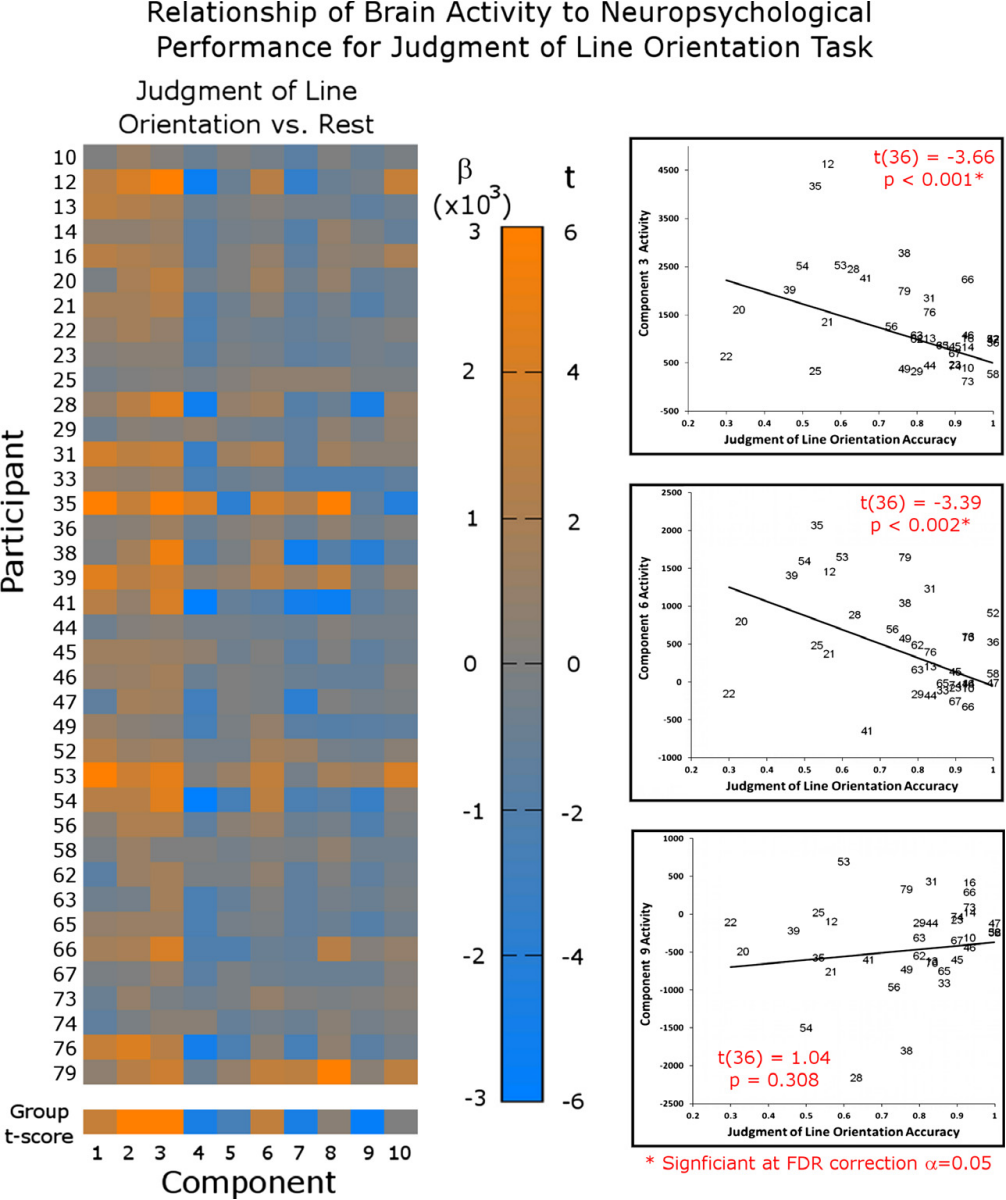
Relationship of brain activity to neuropsychological performance for Judgment of Line Orientation task. (Left) Component *β* values and group *t*-statistics are depicted for each participant (ordinate) and component (abscissas) for the task contrast of Judgment of Line Orientation (JLO) versus Rest, using the same color coding as Figure 2. (Right) Robust linear regression related out-of-scanner JLO performance to component activity for (top) the dorsal visual network C_3_, (middle) the motor network C_6_, and (bottom) right frontoparietal network C_9_. Scatterplots use participant ID code to indicate each participant's accuracy (abscissas) and component/network activity (ordinates). Trendlines depict the robust regression of *β*s to accuracy, along with *t*-statistics and *P*-values testing the hypothesis that slope ≠ 0. Component 3 activity significantly regressed only to JLO accuracy (*P*_uncorrected_ < 0.001), whereas component 6 activity significantly regressed to both JLO accuracy (*P*_uncorrected_ < 0.002) and mean JLO trial duration (*P*_uncorrected_ < 0.017). The regression trendline for component 6 is plotted for mean JLO trial duration (4.78 sec). Component 9 showed significant task-related activity, but the extent of activity did not significantly relate to task (*P*_uncorrected_ < 0.31).

Independent variables that significantly predicted component *β* activity in the stepwise linear regression were then selected as independent variables for a robust linear regression, which was chosen for its ability to minimize the influence of outliers (Wager et al. 2005). Robust linear regression was conducted using Huber M-estimation, which is more resilient to outliers in the response variable (brain activity) than the default Tukey bisquare estimator, and the default tuning constant of 1.345 (Huber and Ronchetti 2009). Stepwise and robust linear regressions were performed using Matlab (code available upon request). Figure 4 depicts the influence of JLO neuropsychological testing accuracy upon brain network recruitment during the JLO fMRI task.

## Results

### Task-dependent activation of intrinsic networks

Figure 3 depicts task-dependent recruitment of intrinsic networks across a broad range of fMRI tasks. Components 1–3 (C_1_, C_2_, C_3_) represent the primary visual, ventral visual, and dorsal visual networks, respectively. All three networks were significantly activated during the viewing of positive or negative visual stimuli (all *t* > 5.7). C_2_ and C_3_ were also activated during the Flashing Checkboard and Judgment of Line Orientation tasks (*t* > 6.6), with lesser activation of C_1_ for these tasks (*t* > 2.8). C_2_ and C_3_ were more active than rest during viewing of MSIT (*t* > 5.7), COWAT (*t* > 7.5), and Word Pair stimuli (*t* > 4.7); conversely, C_1_ was variably activated (or deactivated) for these contrasts.

Component 4 (C_4_) represented default mode network and was consistently less active during task than rest (*t* < −4.0). The depicted exceptions are Left or Right Finger Tapping and viewing of Emotional versus Neutral IAPS stimuli, for which C_4_ was nonsignificant. Component 5 (C_5_) represented bilateral cerebellum and was significantly more active than Rest during Left or Right Finger Tapping (*t* > 4.0), MSIT Congruent or Incongruent stimuli (*t* > 4.5), COWAT Letter or Category tasks (*t* > 4.7) and Flashing Checkerboard (*t* > 4.5). Component 6 (C_6_), representing bilateral primary motor and premotor areas, and was significantly more active than rest for Left Finger Tapping (*t* > 3.6) – but not Right Finger Tapping (*t* > 1.6) – and for 0-Back trials (*t* > 3.4), rating Positive Emotional stimuli (*t* > 3.2), and during Judgment of Line Orientation (*t* > 3.2). C_6_ was less active than 2-Back during Rest (*t* < −6.8) and during 0-Back (*t* < −8.5).

Component 7 (C_7_) represented bilateral temporal cortices and was significantly more active during Right or Left Finger Tapping than Rest (*t* > 5.7) and more active during viewing of Emotional (Positive or Negative) Images than Neutral Images (*t* > 4.9). C_7_ was less active than rest during MSIT trials (*t* < −4.6), 2-Back trials (*t* < −5.7), viewing Positive or Negative Images (*t* < −5.8), and during Flashing Checkerboard or Judgment of Line Orientation tasks (*t* < −2.9). Component 8 (C_8_) consisted of medial frontal, dorsal frontal, and anterior cingulate; C_8_ was less active than Rest for most contrasts, including MSIT (*t* < −4.2), 0-Back and 2-Back (*t* < −3.4), and Positive and Negative images (*t* < −2.7).

Components 9 and 10 (C_9_ and C_10_) represent left and right frontoparietal networks, respectively. C_9_ shows significant activation during the Flashing Checkerboard task (*t* > 4.6) and 2-Back condition (*t* > 4.0), and deactivation during COWAT Letter and COWAT Category (*t* < −5.6) and Judgment of Line Orientation (*t* < −5.4). Conversely, C_10_ shows broad activation across most tasks, including 2-Back (*t* > 6.7), MSIT (*t* > 5.1), and COWAT Letter and Category (*t* > 8.4).

### Individual differences in task-dependent network activation

The n-back task offers many task contrasts, and is thus ideal for demonstrating individual differences in performance. Figure 3 depicts group variation in network recruitment across n-back task conditions.

#### Instructions versus rest

The instructions preceding each task block (during which the words “0-back” or “2-back” instructed participants which task to perform) caused robust group activation of visual networks C_1_ (*t* > 6.6), C_2_ (*t* > 16.9), C_3_ (*t* > 10.6), and C_10_ (*t* > 6.6), with most or all subjects showing task recruitment (87%, 100%, 92%, and 89% of sample for each network, respectively). Components with less activity during Instructions than Rest (C_4_ and C_7_) were less significant (*t* < −5.5) than positively-activated components, but with comparable intersubject variability (76% and 87% of sample).

#### 0-Back versus rest

C_3_ and C_6_ showed overall task-related activation (*t* > 4.0 and 3.4, respectively) with 77% and 74% of sample showing positive activation for this contrast. C_1_, C_4_, and C_8_ were less active during 0-Back than Rest (*t* < −8.8, −6.9, and −3.4, respectively), with strong consistency across subjects (95%, 87%, and 74% of sample showing deactivation).

#### 2-Back versus rest

C_9_ and C_10_ showed task-related activation (*t* > 4.1 and 7.3, respectively) with 71% and 95% of sample showing positive activation for this contrast. C_4_ showed greater activity for rest than task for all participants (*t* < −10.6, 100%). C_1_, C_6_, C_7_, and C_8_ were deactivated for 97%, 76%, 87%, and 89% of the sample (*t* < −9.9, −4.3, −5.7, and −6.5, respectively).

#### 2-Back versus 0-back

C_10_ was most significantly (*t* > 6.7) and consistently (87% of sample) activated component for this contrast, followed by C_9_ (*t* > 4.0, 76% of sample). Greater activity for 0-Back than 2-Back was observed for C_4_ (*t* < −7.9, 92%), C_6_ (*t* < −8.1, 95%), C_7_ (*t* < −9.3, 95%) and visual networks C_1_–C_3_ (*t* < −2.6, range 63–73% of sample).

### Performance-dependent recruitment of intrinsic networks

Figure 4 depicts network recruitment during the JLO fMRI task as a function of performance on the JLO neuropsychological test. Thirty-eight participants had usable data for both sessions. Participant accuracy for the JLO neuropsychological test was highly correlated with accuracy for the JLO fMRI task (*r* = 0.80, *P* < 1 × 10^−8^), indicating that the JLO fMRI task was a successful direct replication of the neuropsychological measure.

Group *t*-tests showed the ventral (C_2_) and dorsal (C_3_) visual networks as having the greatest activity during task than rest (*t* > 8.2 with 92% and *t* > 6.7 with 97% of sample showing recruitment, respectively). Task-related deactivation (greater activity for Rest than JLO) was observed for default mode (C_4_, *t* < −4.7, 82% of sample), temporal (C_7_, *t* < −4.6, 87% of sample), and right frontoparietal networks (C_9_, *t* < −5.5, 87% of sample).

Judgment of Line Orientation task accuracy significantly predicted activity of the dorsal visual network C_3_ (*t*(36) = −3.65, *P* < 0.001) and ventral visual network C_2_ (*t*(36) = −2.41, *P* = 0.021), although the latter does not survive FDR correction for 10 comparisons. In contrast, the motor network C_6_ activity was significantly predicted by JLO accuracy (*t*(35) = −4.20, *P* < 0.001) and mean JLO trial duration (*t*(35) = −2.50, *P* = 0.017). Head coil (8- or 32-channel), age, handedness, education, and sex did not predict brain activity for any components. Finally, the right frontoparietal network C_9_ had the greatest task-related deactivation (*t* < −4.6), but extent of deactivation was unrelated to task performance (*t*(31) = 0.80, *P* = 0.43).

Additionally, poor performers demonstrated greater variance in brain activity than high performers. Participants who performed below the median accuracy (83%; *n* = 20) had significantly greater variability in brain activity (*σ* = 740) than participants with accuracy greater than or equal to 83% (*n* = 20; *σ* = 408; *F*(1, 37) = 10.5, *P* = 0.003). This greater variability – particularly for participants 12, 22, and 35 – could reflect maladaptive strategies for task performance. The strong correlation of JLO performance inside and outside the scanner rules out scanner environment as explaining the difference in brain variability among high and low performers.

## Discussion

We introduce the Cognitive Connectome Project as a methodological framework for translating functional MRI into clinical decision making. We have recruited a sample diverse in age, education, sex, and ethnicity, which we contend is crucial for ecologically valid investigations of individual differences in the neural representation of cognitive variance. Using the same 10 canonical resting-state components, we have replicated many of Smith and colleagues' findings: the three visual networks (C_1_–C_3_) were activated during processing of visual stimuli; the default mode network (C_4_) was consistently deactivated (more active during rest than task) across domains; and left frontoparietal network (C_10_) showed considerable breadth of activation, whereas right frontoparietal network (C_9_) recruitment was largely constrained to working memory tasks. Our independent replication of these meta-analytic findings supports the construct validity of the Cognitive Connectome.

Our replication of Smith et al. (2009) bore some differences that warrant attention. Notably, the motor (C_6_) and executive function networks (C_8_) were minimally recruited by task. We offer two explanations for these findings. First, components derived from ICA of resting-state data may be suboptimal for describing task-elicited activity. For example, ICA of resting-state datasets consistently depict motor networks as bilateral (Beckmann et al. 2005; Wisner et al. 2013), but unimanual tasks asymmetrically recruit contralateral primary motor cortex (Catalan et al. 1998; Gordon et al. 1998). This resulting inhibition of ipsilateral primary motor cortex would diminish the activation magnitude (and significance) of a bilateral motor component. Of note, movements of the nondominant hand are less asymmetric (more bilateral) than dominant hand movements (Hayashi et al. 2008); accordingly, left-hand finger tapping in our predominantly right-handed sample led to greater C_6_ recruitment (*t* > 3.1) than right-hand finger tapping. Likewise, the executive function component consists of orbitofrontal, ventromedial, and dorsolateral prefrontal networks, which have dissociable roles in emotional and cognitive processing (Robinson et al. 2014). Thus, the merging of these distinct networks into a single component may be too general to adequately capture the complexity that we as a field refer to as executive function. Future work will explore task-dependent variation in the neural representation of executive function.

Second, in an effort to minimize participant burden by limiting MRI scanning to two 1-h sessions, the Cognitive Connectome represents some cognitive domains using a single task. This over-generalization undoubtedly leads to experiment-specific nuances in brain activation that are evident in Figure 2. For example, the MSIT was chosen from roughly a dozen well-characterized attentional conflict tasks (Oddball, Flanker, Stroop, Simon, etc.) for its standardized administration and incorporation of multiple forms of attention. Consequently, MSIT elicited greater activation of C_2_ and C_3_ than Smith and colleagues had reported for attentional conflict tasks. We attribute this unexpectedly strong visual activity to the visuospatial properties of MSIT's horizontally arranged numerical stimuli. Incorporating multiple diverse attentional tasks (e.g., tasks using verbal stimuli) may have resolved this discrepancy; however, this would have required a third MRI session, resulting in greater participant attrition. We again stress the importance of group-level meta-analyses and conceptual replications to dissociate neural activity globally involved in a cognitive domain from activity that is specific to a given neuroimaging paradigm.

Our analysis of intersubject variability (Fig. 3) shows that brain networks may show significant task recruitment for a group yet still vary considerably across individuals. Although we replicated the group finding of greater left frontoparietal activity during 2-Back versus 0-Back conditions (*t* > 6.7), 5 (13%) of 38 participants showed left frontoparietal deactivation (i.e., greater activity for 0-Back than 2-Back). As *t*-statistics reflect both magnitude and standard deviation of activation, a high *t*-score could result from a few subjects with exceptionally high-component activations, subjects with moderate but consistent activations, or both. For the tasks discussed, the visual networks (when showing significant group activation) demonstrated greatest intersubject consistency in activation (range 67–100% of sample; mean [SD] = 87% [11%]), and the default mode network showed comparable deactivation (range 74–100%, mean [SD] = 85% [10%]).

We further demonstrated that for the Judgment of Line Orientation task, our most direct replication of a neuropsychological test, task performance scaled with recruitment of some (but not all) networks. Dorsal visual network was most significantly recruited by task, which is consistent with its role in judging visuospatial orientation (Buchel et al. 1999; Sack et al. 2002). Dorsal visual network activity negatively regressed to JLO accuracy, which we interpret as high performers requiring less network recruitment during task. Conversely, task recruitment of the motor network negatively regressed to both JLO accuracy and mean JLO trial duration. Our finding is consistent with reports of motor network involvement in visuospatial processing (di Pellegrino and Wise 1993; Vingerhoets et al. 2002; Rushworth et al. 2003; Lamm et al. 2007) as well as reports of greater motor network activity with prolonged duration of motor response (Yarkoni et al. 2009).

Conversely, networks such as right frontoparietal and default mode showed task-related decreases in activity, but these task-related changes in activity were unrelated to performance. Although changes in default mode network connectivity have been reported for numerous neurologic and psychiatric conditions including epilepsy (Liao et al. 2010; James et al. 2013), schizophrenia (Hasenkamp et al. 2011; van Lutterveld et al. 2014), Alzheimer's disease (Greicius et al. 2004; Jones et al. 2012), and depression (Guo et al. 2013; Sambataro et al. 2013), the extent to which these changes encode symptom severity or cognitive impairment is unknown. We provide a framework by which task-dependent changes in default mode can be associated to symptom in clinical populations.

### Caveats and limitations

The Cognitive Connectome Project was designed to sample cognition as broadly as possible while minimizing participant burden. Furthermore, fMRI tasks were designed with the limitations of clinical populations taken into consideration. For these reasons, fMRI tasks were optimized to give greatest detection power in the shortest timeframe, as is typical of block designs (Hagberg et al. 2001). For example, working memory would be more completely characterized by an n-back task with parametrically varied loads (i.e., 0-, 1-, 2-, and 3-back conditions). We instead opted for an n-back task with only 0-back and 2-back conditions, as the 1-back condition is trivial for healthy adults, and the 3-back condition is too difficult for cognitively impaired patients. Nonetheless, numerous studies have used the contrast of 2-back versus 0-back to map working memory function (Owen et al. 2005), and this strategy has recently been adopted by the Human Connectome Project (Barch et al. 2013).

Similarly, many of the Cognitive Connectome Project's fMRI tasks use a fixation cross as a low-level control condition, whereas high-level control conditions are generally more preferable because they capture task-irrelevant cognitive processes (Price et al. 2005). For example, 2-back trials require multiple cognitive processes (such as motor execution, visual processing, and working memory) which could not be fully dissociated with comparison to fixation trials; thus, 0-back trials are included to control for 2-back cognitive demands that are unrelated to working memory. While it would be ideal to include high-level control conditions for all tasks (such as nonsense words for the COWAT task, passive viewing of a motor cue without response for the finger tapping task, etc.), the inclusion of these additional high-level controls would have dramatically inflated task duration by an estimated 30–40% – necessitating an additional hour of MRI testing (and exacerbating participant dropout while diminishing sample size) or reducing the number of cognitions studied. We instead opted to use a fixation cross as a standard low-level control across all tasks (including tasks with high-level controls, such as the n-back), thus improving our ability to make inferences between tasks.

Our usage of low-level baseline conditions was also motivated by the eventual translation of the Cognitive Connectome Project into clinical decision making. One could argue that a low-level baseline condition is preferable for surgical preplanning (such as mapping a language network), as it would capture all aspects of language (from recognizing a letter from its visual features to retrieving a word's semantic associations to accessing the word's phonetic properties). Mapping language networks using high-level baseline condition could miss some of these more basic cognitive processes, potentially resulting in postsurgical deficits. This argument is supported by recent evidence that the COWAT task with low-level baseline condition more robustly activated language-associated brain regions than tasks with high-level baseline conditions such as the sentence completion task and noun–verb association (Zaca et al. 2013).

Finally, no single fMRI task can fully capture the complexity of an entire cognitive domain. Our selection of the MSIT for modeling anterior cingulate activity during conflict processing was largely motivated by MSIT's standardized administration. Conflict modeling would ideally be modeled using multiple fMRI paradigms (such as Stroop, Ericksen flanker, and Simon tasks) with both auditory and visual administration. Additionally, every fMRI task design may elicit unique task-specific activations. For these reasons, meta-analyses will always be valuable for mapping commonalities in cognition-dependent neural recruitment.

### Future directions

The clinical success of the Cognitive Connectome rests upon its ability to map normative variance in cognition, particularly with regard to diverse demographic variables. The strongest demographic influence on neuropsychological performance is age, followed by education. Other factors such as sex and ethnicity have negligible influence upon cognitive measures after controlling for these two variables. Most of the Cognitive Connectome's neuropsychological instruments have normative values for age only, although some instruments (particularly those measuring language and executive function) have normative values for both age and education. The normative scoring for these instruments typically bins adult participants into class intervals of 8–10 years. (Children are typically binned into narrower intervals of 2–4 years.) Based on our findings of stable brain–behavior relationships for 30–40 healthy adults per task, we estimate that – in order to make clinical inferences for a single patient – we would need roughly 30–40 healthy adults of that participant's age ±5 years. In other words, an estimated 30–40 participants per decade of life, or 210–280 participants aged 20–90, would be needed for the Cognitive Connectome to reach its full translational potential.

## Conclusions

We introduce the Cognitive Connectome as a tool for comprehensively mapping the neural basis for normative variance in cognition, behavior, and personality. The Cognitive Connectome represents a novel collaboration between neuroimaging and clinical neuropsychology to mutually inform each field, and in doing so provide a broader understanding of cognition and its neural representations. We believe our efforts to capture a diverse sample representative of the general population is crucial for neuroimaging's clinical translation and its future use in personalized medicine.

## Supporting Information

**Figure S1.** Extracting component activity timecourses.

**Appendix S1.** Description of Neuropsychological Tests and Neuroimaging Tasks.
