# Supplementary figures and images for: Task-dependent recruitment of intrinsic brain networks reflects normative variance in cognition
Source: Brain Behav. 2014 Jul 9;4(5):650–64. doi: 10.1002/brb3.243 (PMC4107383; doi:10.1002/brb3.243)

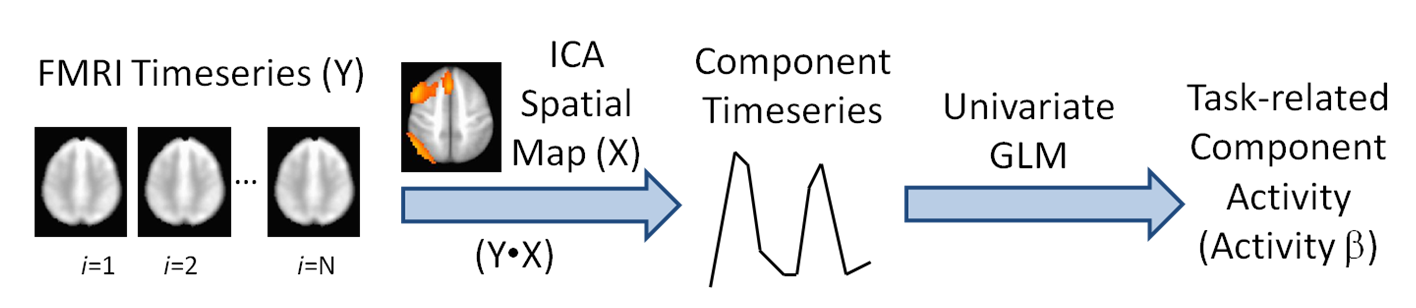

Supplement: Supplementary file 1 — Figure S1. Extracting component activity timecourses. [file brb30004-0650-SD1.png]
