# Supplementary material for: Task-dependent recruitment of intrinsic brain networks reflects normative variance in cognition
Source: Brain Behav. 2014 Jul 9;4(5):650–64. doi: 10.1002/brb3.243 (PMC4107383; doi:10.1002/brb3.243)
Supplement: Supplementary file 2 — Appendix S1. Description of Neuropsychological Tests and Neuroimaging Tasks. [file brb30004-0650-SD2.docx]

**Appendix S1.** Description of Neuropsychological Tests and Neuroimaging Tasks.

**Neuropsychological Measures**

*Grooved Pegboard*: The Grooved Pegboard is a timed, bilateral manipulative dexterity test. The box consists of a field of 25 holes with randomly positioned slots. Pegs with a key along one side must be rotated to match the hole before they can be inserted. Participants are allowed to use only one hand, and are timed on their speed with which they can correctly place the keys in the slots. Timed measures are taken for dominant and non-dominant hands (Lezak 1995). This test was administered as per standardized instructions.

*Halstead-Reitan Finger Tapping Test*: The Halstead-Reitan Finger Tapping Test is a timed, bilateral fine motor speed test. It was originally called the Finger Oscillation Test (FOT) and was part of the Halstead test battery (Halstead 1947). Using a specially adapted tapper and counter, the examinee is instructed to tap as rapidly as possible, using the index finger. Seven trials of 10 seconds each are given for each hand. This test was administered as per standardized instructions.

*Benton Judgment of Line Orientation Test*: The Benton Judgment of Line Orientation Test is a neuropsychological measure widely used to assess visuospatial processing (Benton, et al. 1983). This measure asks participants to match two line segments against 11 lines of differing angles (0^o^, 18^o^, 36^o^ … 162^o^, 180^o^). A total of 30 items are presented, with each item consisting of two line segments. Difficulty is graded across items, with shorter line segment lengths and closer angles between segments used to increase difficult. The total score is the number of correct items (0-30).

This test was administered as per standardized instructions.

*Rey-Osterrieth Complex Figure Tes (ROCF)*: ROCF test is an anterograde visual learning and memory task (Osterrieth 1944). In this test, the participant is asked to copy a complex geometric figure. Participant accuracy in reproducing the figure is a measure of visuospatial ability. After a subsequent delay, the participant is asked to reproduce the figure from memory as a measure of visual memory. The Copy condition (and not the Memory condition) was administered as per standardized instructions.

*Test of Everyday Attention (TEA)*: TEA is an eight subtest test battery designed to assess various attentional components (Robertson, et al. 1996). TEA subscales include Elevator Counting (sustained auditory attention), Elevator Counting with Distraction (selective attention and working memory), Visual Elevator (a visual analog of the Elevator Counting subtest; sustained visual attention), and Elevator Counting with Reversal (attentional switching and auditory working memory). TEA was administered as per standardized instructions.

*WAIS-IV Digit Span*: The WAIS-IV version of the Digit Span consists of three subtests: Digit Forward, Digit Backward, and Digit Sequencing (Wechsler 2008). Each subtest consists of random number sequences that the examiner reads aloud at a rate of one digit per second. Digit Forward taps basic attentional skills by requiring the participant to simply repeat the string of digits. Digit Backward requires both verbal attention and working memory by having the participant repeat the string of digits in reverse order. Digit Sequencing poses additional working memory load by requiring the participant to repeat the numbers in order from lowest to highest value. This test was administered as per standardized instructions.

*WMS-III Spatial Span:* The WMS-III version of the Spatial Span is a nonverbal analog of the Digit Span (Wechsler 1997). Instead of remembering a digit sequence, participants are asked to remember a briefly presented spatial sequence. Like the digit span, the spatial span taps basic attention skills and working memory, but without the confound of verbal ability. Spatial Span consists of a Spatial Forward subtest (in which the participant repeats the spatial sequence) and Spatial Reverse subtest (in which the participant repeats the spatial sequence in reverse order). This test was administered as per standardized instructions.

*D-KEFS Verbal Fluency Test*: The Verbal Fluency Test (also known as the Controlled Oral Word Association Task) assesses verbal fluency, or the ability to organize information to produce a series of related words in an efficient manner (Benton and Hamsher 1989). There are two main types of verbal fluency: letter and category. Letter fluency involves listing as many words as possible beginning with a target letter (such as “A”) within a finite period of time, while category fluency requires the participant to list as many words within a given category (such as “animals”). D-KEFS administers these tests as 3 conditions: Condition 1 (Letter Fluency), Condition 2 (Category Fluency) and Condition 3 (Category Switching). This test was administered as per standardized instructions.

*Boston Naming Test*: The Boston Naming Test is a measure of object naming (Kaplan, et al. 1983). This assessment requires the participant to produce the common name of up to 60 ink drawings. The drawings progressively increase in difficulty, beginning with such drawings as “tree” and “pencil” and terminating with objects such as “trellis” and “abacus.” If a participant is unable to spontaneously produce the name of the object, s/he is then given a semantic clue; phonemic cues are provided if the semantic cue is not assistive. This test was administered as per standardized instructions.

*California Verbal Learning Test- Second Edition (CVLT-II)*: Anterograde verbal learning and memory was assessed using the CVLT-II (Delis, et al. 2000). The CVLT-II is a verbal list-learning task that contains a list of 16 semantically clustered words, presented in a random array, that are presented over five successive learning trials. For each trial, the list of words is read at a rate of one word every second. The participant is then asked to list as many words as possible, in any order. Following presentation of a distracter list and a 20-minute delay, participants are asked to recall the original list of words. This is followed by a recognition task, containing the original 16 items as well as 16 semantically related and unrelated words. This test was administered as per standardized instructions.

*WMS-IV Verbal Paired Associates Task:* The WMS-IV Verbal Paired Associates Task (also known as the Paired Word Associates task) is a measure of verbal memory (Wechsler 2009). The participant is presented 14 word pairs, one at a time, and is asked to memorize the words. Memory is tested by showing the participant one word from each pair, and asking the participant to recall the other word. This process is repeated 4 times using the same word list and cue words. Participants should progressively improve their memory of the 14 word pairs. This task also includes a Delayed Recall condition (in which participants are retested on the cue words 10-20 minutes after the 4th administration) and a Recognition task (in which participants are given the 20 word pairs and 20 distractors and must identify the previously memorized word pairs). This test was administered as per standardized instructions.

*Brief Visuospatial Memory Test - Revised (BVMT-R):* The BVMT-R is a tool to assess visual perception and visual memory(Benedict, et al. 1996). Participants are shown a page of 6 graphical designs for ten seconds, then asked to reproduce the figures they saw on a sheet of paper. This is repeated 3 times. After an approximately 25 minute delay, the participant is asked to again reproduce the previously seen figures. Finally, the participant is shown the 6 viewed figures and 6 new figures, and asked to pick the viewed figures from the new ones. This test was administered as per standardized instructions.

*Temporal (delay) discounting*: A temporal (or delay) discounting task was administered to assess reward processing (Bickel, et al. 2009). Participants chose between receiving hypothetical money today (with values ranging from $1-$1000) of a hypothetical sum of $1000 at a future delay (from one week to 25 years). Seven delay periods were used: 1 day, 7 days, 1 month, 6 months, 1 year, 5 years, and 25 years. Delays were presented in a block design with 6 trials per delay. The first trial for each delay offered an immediate value of $500; each choice of immediate or delayed reward caused the next trial’s immediate value offered to decrease or increase (respectively). This task was used to determine each participant’s “indecision point” – the point where the participant has equal preference for immediate or delayed reward – which is a measure of impulsivity.

*D-KEFS Tower Test.* The D-KEFS Tower (of Hanoi) taps several areas of executive function, including planning, inhibition of preservative or impulsive responses, rule learning, and set maintenance (Delis, et al. 2001). The D-KEFS Tower consists of disks of varying diameters and three upright pegs; the disks have holes in their centers so that they may be stacked upon the pegs. For each trial, the administrator arranges the disks on the pegs into a predetermined starting position and presents a picture illustrating a predetermined ending position. The participant is instructed to move disks one at a time until the physical tower matches the depicted tower, with the rule that wider disks can never rest atop narrower disks. The participant is also instructed to build the depicted tower in the fewest number of moves possible. Number of moves to completion, item-completion time, accuracy, number of rule violations, and time until initiating first move are recorded. This test was administered as per standardized instructions.

*D-KEFS Color-Word Interference Test:* The Color-Word Interference Test, colloquially known as the Stroop task (Golden 1978), is a commonly used measure of executive functioning for both clinical and research purposes. This test in part measures response inhibition but is also a measure of complex concentration, selective processing, and attention. The D-KEFS version of this test consists of 4 conditions. In condition 1, the participant sees a page with patches of color and must name each color as quickly as possible. In condition 2, the participant sees a page with names of colors and must read them aloud as quickly as possible. In condition 3, the participant sees pages of color names printed in an incongruent color (such as “red” printed in green) and must name the color of each font. In condition 4, the participant sees a page similar to condition 3 but with some words surrounded by a box; the participant performs as per condition 3 except for boxed words, for which participant must read the word and not name the color. This test was administered as per standardized instructions.

*Wisconsin Card Sorting Test*: The Wisconsin Card Sorting Test (Berg 1948) is an assessment of abstraction ability and the ability to shift and maintain cognitive strategies for categorization. The participant is shown 128 stimulus cards (each depicting a shape, number, and color, such as 3 yellow stars) one at a time and is required to match each card to one of four key cards. After each match, the participant is told whether they are correct. After a predetermined number of successful sequential matches, the matching criteria (color, shape, or number) shifts, requiring the participant to learn the new matching condition. The test requires planning, organized searching, the use of environmental feedback to inform decisions, and restriction of impulsive responding. Generated scores include number of total errors, number of perseverative errors, failure to maintain set, and number of categories completed. This test was administered as a computerized instrument as per standardized instructions (Heaton and Staff 2008).

*Booklet Category Test*: The Booklet Category Test is based upon the original Category Test conceptualized from categorization strategies from brain-injured individuals (Halstead and Settlage 1943). The Booklet Category Test consists of 208 items, divided into seven different subtests. For each item, the participant is asked to identify the number (one, two, three, or four) suggested by the stimulus item. Subtests utilize different categorization schemes, which the participant deduces from feedback on his/her performance. This test was administered as per standardized instructions.

*D-KEFS Trail Making Test*: The Trail Making test is an assessment of simple and complex visual scanning and motor ability originally part of the Army Individual Test Battery (Lezak 1995). This test traditionally has two components: Trails A and Trails B. Trails A is a simple task of visuomotor scanning and sequencing that requires the participant to connect consecutively numbered circles (1-2-3-4-5-6…) as rapidly as possible. Trails B is a test of complex visual sequencing, motor speed, and agility that requires the participant to alternately connect numbers and letters in sequence (A-1-B-2-C-3…). Trails B is a more demanding task in that it has more interfering visual stimuli, demands consistent set-shifting, and requires more skilled search and motor skills (Gaudino, et al. 1995). While the Trail Making Test is typically administered as two subtests (A and B), D-KEFS administers the Trail Making Test as five separate subtests for finer evaluation of cognitive and motor skills. (Test 1-5). This test was administered as per standardized instructions.

*D-KEFS Proverb Test:* The Proverb Test consists of eight sayings presented in two conditions: (1) free inquiry, and (2) multiple choice (Delis, et al. 2001). The first condition asks the participant to describe the meaning of a common saying, such as “No good deed goes unpunished”. The second condition presents the same proverbs, but also provides multiple-choice answers explaining the proverb, from which the participant chooses the best answer. This test measures the participant’s ability to extract and synthesize verbally-presented information. This test was administered as per standardized instructions.

## Neuroimaging Tasks

*Resting-state*. Each fMRI session began with a resting-state scan. For this scan, participants were presented a fixation cross and received the following instructions: “For this task, we will measure brain activity in the absence of task. Please look at the cross. It is there to keep your eyes from wandering. Also, please try to keep your mind from wandering – try not to think of anything specific. Just relax and look at the cross.” Each scan lasted 7min 30s.

*Flashing Checkerboard task*. The flashing checkerboard task is a passive viewing task that uses rapidly alternating high-contrast visual stimuli (for this task, a checkerboard whose black and white squares alternate color at ~4Hz frequency) to map neuronal activation of the visual cortex (Blamire, et al. 1992; Schneider, et al. 1993). This task robustly activates visual cortex and is frequently used as a control task for tasks with greater cognitive demand; however, it has also been used to demonstrate changes in visual cortex activity due to stimulus properties such as luminance (Mohamed, et al. 2002) and color (Kruger, et al. 1998). The Flashing Checkerboard and Finger Tapping tasks were presented as a single paradigm for the first five pilot participants; these tasks were separated for subsequent participants. This task consisted of four 18s blocks of checkerboard alternating with five 10s blocks of visual fixation, for a total duration of 2min 2s.

*Finger Tapping task*. The finger tapping task is widely used in both normal and pathologic populations to elicit motor activation; finger tapping tasks vary by whether they are paced and by complexity of the task (i.e. multifinger, sequential, bimanual). Finger tapping elicits response within the motor cortex, which includes primary sensorimotor cortex, supplementary motor area, basal ganglia, and cerebellum with other areas of activation also elicited depending on task dynamics (Witt 2008). For this task, participants underwent 18s blocks of finger tapping using their left or right index finger (3 blocks per condition; 6 total). Block condition was indicated non-verbally by an arrow pointing left or right. Participants were instructed to make as many keypress responses as they could for the duration of the arrow display. Blocks were separated by nine 10s Rest blocks, consisting of a fixation cross. The first five pilot participants underwent a task combining Flashing Checkerboard and Finger Tapping tasks; total task time was 2min 58sec for remaining participants.

*Judgment of Line Orientation (JLO) task*. The fMRI variant of the JLO task closely replicated the Benton Judgment of Line Orientation test. 15 trials of varying difficulty were selected from the Benton JLO Form H and modified for the MRI environment as follows. Although the Benton JLO requires participants to match two line segments against 11 lines of equidistant angles (0^o^, 18^o^, 36^o^, 54^o^, 72^o^, 90^o^, 108^o^, 126^o^, 144^o^, 162^o^, or 180^o^), the fMRI version used a button box that permitted only 8 possible responses (corresponding to the 8 fingers, excluding thumbs). Stimuli were adapted by removing the 0^o^, 90^o^, and 180^o^ response choices. Each trial presented two line segments in 2 of 8 possible angles (18^o^, 36^o^, 54^o^, 72^o^, 108^o^, 126^o^, 144^o^, or 162^o^) at the top of the screen, and the eight possible matches at the bottom of the screen. Each trial lasted 15 seconds, ending when the participant made two matches (during which a fixation cross was presented until the start of the next trial. Accuracy was calculated as the total number of items (trials) for which both line segments were correctly matched. For the first five pilot participants, trials consisted of only the two line segments (and not the 8 matching lines); pilot participants reported high task difficulty, warranting the addition the guidelines to facilitate performance.

*n-back task*: The *n*-back task (Cohen *et al.,* 1994) is a task requiring attention and working memory. In general, the participant views a sequence of stimuli and responds whenever the current stimulus matches the stimulus presented “*n*” trials ago, where *n* is typically 1-3. Specifically for this task, participants viewed a pre-randomized sequence of uppercase letters (A-E), with each letter presented for 1,250ms with a 250ms interstimulus interval. Participants underwent a 0-back condition (responding whenever the letter “A” appeared) and a 2-back condition (responding whenever the current letter matches the letter presented two trials ago, as in “C-B-C”). The first five pilot participants (Subjects 001-007) underwent a task version consisting of alternating 90s blocks of 0-back and 2-back trials (3 blocks each; 6 total), with each block preceded by a 6s Instruction block (consisting of the words “0-back” or “2-back”) and followed by 20s Rest block (consisting of a fixation cross); this paradigm lasted 11min 36sec. Pilot analyses showed comparable results when using only two blocks or all three blocks of each task condition, leading to a task redesign. Subsequent participants underwent *n*-back task with alternating 40s blocks of 0-back and 2-back conditions (4 blocks each; 8 total), preceded by a 5s Instruction block and proceeded by a 15s Rest block; total task time was 7min 50sec. *n*-back has been shown to reliably activate bilateral dorsolateral prefrontal cortex and bilateral superior parietal cortex across many stimulus types (Owen, et al. 2005).

*Letters and Category Verbal Fluency task (Controlled Oral Word Association Task; COWAT)*: The fMRI COWAT variant consisted of ten 15s task blocks each proceeded by a 15s Rest block consisting of a fixation cross. When presented a letter during the task block, participants were instructed to covertly generate as many words beginning with that letter for the duration of the task block; when presented a category, participants were instructed to generate words belonging to that category. Task blocks consisted of letter/category presentations in this order: “R”, “Plants and Flowers”, “P”, “Clothing”, “W”, “Foods”, “S”, “States”, “J”, “Jobs”. Total scan time was 5min.

*Verbal Paired Associates Task*. The fMRI variant of the Paired Associates Task consisted of 10 trials, with each trial consisting of two words displayed for 10s. Participants were asked to memorize all 10 word pairs (20 words total). The experiment began and ended with a 10s Rest period for a total scan time of 2min. Immediately after the scan (before the next scan), the experimenter conducted a cued recall test by reading the first word from each word pair and asking the participant to provide the corresponding word pair item. The first five pilot participants conducted a free recall prior to the cued recall; the free recall was discontinued for subsequent participants after reports that it interfered with the cued recall.

*Encoding and recalling affective pictures*: The International Affective Picture System (IAPS) is a collection of 1,000 color images normed for valence, arousal, and dominance (Bradley and Lang 2007). IAPS images are regularly used in fMRI research of emotional processing, with studies suggesting that viewing emotionally evocative pictures (both positive and negative) results in activation of orbitofrontal and lateral prefrontal cortex, anterior cingulate, insula, amygdala, and other limbic regions (Aldhafeeri, et al. 2012; Grimm, et al. 2006; Wright, et al. 2008). The first five pilot participants (001-007) underwent an emotional viewing paradigm in which they viewed 90 IAPS stimuli (30 positive, 30 neutral, and 30 negative, as determined from normative ratings). Each stimulus was presented for 5s with 2-6s intertrial interval. Participants rated each image during its presentation as positive, neutral, or negative via buttonpress. Outside of the scanner (~20min post-viewing), participants then underwent free- and cued-recall of the 90 images.

Due to poor recall performance in these pilot participants, the IAPS paradigm was separated into two paradigms: an Encoding paradigm identical to the one described above but using 45 images (15 positive, 15 neutral, and 15 negative) and a Recognition paradigm administered in-scanner ~20min post-viewing. The Recognition paradigm displayed all 90 images (presented for 5s with 2-6s intertrial interval); participants were instructed to respond via “yes/no” buttonpress whether each image had been previously seen. The 45 stimuli selected for the Encoding paradigm and the order of stimuli presented during Cued Recall were counterbalanced across participants.

*Iowa Gambling task*: The Iowa Gambling task (Bechara, et al. 1997) is a widely used instrument assessing decision making and reward. Participants begin with a hypothetical pool of $2000 and draw cards from 4 decks. Each card causes the participant to gain or lose hypothetical money, with the magnitude of gain or loss varying between decks. The test assesses participants’ ability to predict which deck gives the greatest gains with the fewest losses, as well as gauge participants’ responses to reward and punishment. Participants drew 100 cards, with each deck containing only 50 cards. Intertrial interval and total scan time varied due to the self-paced nature of this task.

*Tower of London task*: Similar to the Tower of Hanoi task, the Tower of London task measures rule-based planning and working memory (Berg and Byrd 2002; Owen, et al. 1990). In 2000, the Tower of London was adapted for fMRI use (Lazeron, et al. 2000). Whereas the Tower of Hanoi task has different sized discs atop 3 pegs and the rule that larger discs cannot be placed atop smaller discs, the Tower of London has colored balls and 3 pits of different depths, with the rule that a given pit can only hold one, two, or three balls. This variant of the tower task provides a different approach for measuring the same cognitive construct of planning, without the confound of test-retest learning effects. Aside from this variant rule, the two Tower tasks are performed identically. This task has been studied both in control and clinical populations and has been associated with recruitment of bilateral dorsal prefrontal-parietal-striatal network (Boghi, et al. 2006; van Tol, et al. 2011; Wagner, et al. 2006).

The Tower of London task consisted of 8 “Easy” trials (solvable in 2 moves), 8 “Medium” trials (solvable in 3 moves) and 8 “Hard” trials (solvable in 4 moves). The first five pilot participants (001-007) underwent a variant in which Easy, Medium, and Hard trials consisted of 2, 4, and 6 move solutions; however, this paradigm was simplified as described due to poor participant performance and reports of frustration. Participants were instructed to plan their movements during the first 5s of each trial (during which the word “Plan” appeared at the top of the screen and buttonpresses would not move the balls). Participants could begin moving stimuli once “Plan” was replaced by “Execute”; however, participants were encouraged to keep planning as long as needed and only move stimuli once the solution had been determined. Trials had an intertrial interval of 8s, during which participants viewed a fixation cross. Total scan time varied due to the self-paced nature of this task.

*Multi-Source Interference Task (MSIT*). The Multi -Source Interference Task (MSIT) was developed as a standardized fMRI task to robustly activate cognitive subdivisions of the dorsal anterior cingulate cortex (dACC) (Bush, et al. 2003). MSIT combines elements of multiple cognitive interference tasks (i.e., Eriksen Flanker, Simon effect, and Stroop) with decision making, novelty detection, attentional control, error detection, and other factors thought to activate dACC. MSIT was reported to reliably activate dACC in individual subjects while also coactivating dorsolateral prefrontal cortex (DLPFC), premotor, and parietal areas. Furthermore, MSIT conflict trials were reported to produce a mean interference effect of 281 ms when compared against control trials without stimulus or response conflict. Both factors represented a significant advance in the functional neuroimaging of cognitive control given that most standard interference tasks, including the Stroop, are useful only for group analyses and have interference effects generally below 100 ms (Stins, et al. 2005). Based upon these task properties, the MSIT has been widely adopted to investigate dACC activation in clinical samples including schizophrenia (Harrison, et al. 2007), obsessive compulsive disorder (Yucel, et al. 2007), ADHD (Brown, et al. 2010; Bush, et al. 2008), post-traumatic stress disorder (Shin, et al. 2011b), and addiction (Gruber, et al. 2012; Harding, et al. 2012), as well as individuals at risk for PTSD (Shin, et al. 2011a).

**Additional Demographic tests (Self-report inventories)**

*Big Five Inventory*: The Big Five Inventory (John, et al. 1991; John, et al. 2008) assesses five traits of personality: Openness, Conscientiousness, Extraversion, Agreeableness and Neuroticism. Validation studies have shown these five scales to be distinct and independent aspects of personality.

*Beck Depression Inventory:* The Beck Depression Inventory is an instrument for measuring severity of depressive symptoms, consisting of 21 multiple choice questions assessing symptoms such as hopelessness, irritability, guilt, fatigue, weight loss, and anhedonia. (Beck and Steer 1984)

*Childhood Trauma Questionnaire*: The CTQ is a 28-item self-report inventory that describes early life trauma experienced by the participant (Bernstein, et al. 1994). It assesses emotional abuse, physical abuse, sexual abuse, emotional neglect, and physical neglect.

*State-Trait Anxiety Inventory*: The STAI is a 40 question inventory for measuring state anxiety (anxiety the participant currently experiences) and trait anxiety (long-standing, general anxiety) (Spielberger, et al. 1983).

*The Leisure Time Exercise Questionnaire*: The LTEQ is a self-explanatory, four-item instrument for quickly assessing participant’s leisure time exercise habits (Godin and Shephard 1985) .

*Emotion Regulation Questionnaire*: The ERQ is a 10 question inventory measuring emotional experience (how strongly the participant reports feeling emotions) and emotional expression (how often the participant reports showing emotions). The ERQ assesses both positive and negative emotions, and also assesses the participants’ reappraisal and suppression of emotion (Gross and John 2003).
